# Polarity of c-di-GMP synthesis and degradation

**DOI:** 10.1093/femsml/uqad014

**Published:** 2023-04-05

**Authors:** Vanessa Kreiling, Kai M Thormann

**Affiliations:** Justus-Liebig-Universität, Institute of Microbiology and Molecular Biology, Heinrich-Buff-Ring 26, 35392 Giessen, Germany; Justus-Liebig-Universität, Institute of Microbiology and Molecular Biology, Heinrich-Buff-Ring 26, 35392 Giessen, Germany

**Keywords:** *Caulobacter*, *Pseudomonas*, *Shewanella*, attachment, cell cycle, flagella, pili

## Abstract

The bacterial cell pole has long been recognized as a defined compartment for enzymatic activities that are important or even vital for the cell. Polarity of diguanylate cyclases and phosphodiesterases, enzymes that synthesize and degrade the second messenger c-di-GMP, has now been demonstrated for several bacterial systems. Here we review these polar regulatory systems and show how the asymmetry of c-di-GMP production and turnover in concert with different modes of activation and deactivation creates heterogeneity in cellular c-di-GMP levels. We highlight how this heterogeneity generates a diverse set of phenotypic identities or states and how this may benefit the cell population, and we discuss reasons why the polarity of c-di-GMP signaling is probably widespread among bacteria.

## Teaser

Polarly localized production of the secondary messenger c-di-GMP results in asymmetry of c-di-GMP levels after cell fission and the formation of phenotypically distinct subpopulations of cells.

## Introduction

### c-di-GMP signaling

The role of the cyclic dinucleotide molecule bis-(3′–5′)-cyclic diguanylic acid (c–di–GMP) as an important secondary messenger is well recognized for numerous bacterial species. c-di-GMP is synthesized from two Guanosine-5'-triphosphate (GTP) molecules by diguanylate cyclases (DGCs) whose catalytic sites harbor a signature harbor a signature glycine-glycine-aspartic acid-phenylalanine (GGDEF) motif (Chan et al. 2004; Wassmann et al. 2007; Schirmer 2016). Degradation of c-di-GMP is carried out by specific hydrolases that are characterized by conserved glutamic acid-alanine-leucine (EAL) or histidine-aspartic acid/glycine-tyrosine-proline (HD-GYP) motifs. While the latter convert c-di-GMP directly into two GMP molecules (Bellini et al. 2014), EAL-domain proteins linearize c-di-GMP to pGpG (Christen et al. 2005; Barends et al. 2009; Tchigvintsev et al. 2010), which is then further cleaved into two GMP molecules, e.g. by the exoribonuclease Orn (Cohen et al. 2015; Orr et al. 2015). GGDEF, EAL, and HD-GYP domains may occur as stand-alone proteins, but usually they are part of multidomain proteins that additionally harbor various sensor or output domains. Quite frequently, GGDEF and EAL domains are combined in a single hybrid protein, where one or also both domains can be catalytically active (Seshasayee et al. 2010). To exert its regulatory function, c-di-GMP has to bind to designated effector molecules, of which numerous have been identified. Among these are receptors that act as transcriptional regulators and proteins with PilZ or MshEN domains that function as c-di-GMP-dependent adaptors, e.g. in polysaccharide production and motility. Furthermore, there are proteins with degenerate EAL or GGDEF domains, mRNA riboswitches, and catabolic enzymes (Amikam and Galperin 2006; Sudarsan et al. 2008; Hengge 2010; Krasteva et al. 2012; Roelofs et al. 2015; Chou and Galperin 2016; Wang et al. 2016; Schumacher et al. 2022).

c-di-GMP regulation affects various cellular processes in bacteria. Most commonly, c-di-GMP levels are implicated in the switch between planktonic and sessile/biofilm life style, but also the cell cycle, cellular morphology, and pathogenicity are subject to regulation by c-di-GMP in diverse species (Hengge 2009; Römling et al. 2013; Jenal et al. 2017; Valentini and Filloux 2019). Notably, most bacteria possess a plethora of enzymes, which are putatively involved in c-di-GMP production and turnover and which may respond to different cell-internal or -external signals. In addition, this array of potential c-di-GMP inputs is usually accompanied by multiple potential effector and output systems in the cell. To enable an appropriate specific local response to c-di-GMP, despite the input/output complexity, it was proposed that bacteria dynamically combine modes of global and local signaling (Römling et al. 2013; Hengge 2021). Mechanisms of global signaling include, e.g. temporal control of signaling component production and differing affinities of the corresponding effectors, while local c-di-GMP signaling can be realized by organizing the appropriate components into spatially confined modules (Hengge 2021).

### Heterogeneity in c-di-GMP signaling

Genetically identical populations of bacteria have long been appreciated to exhibit phenotypic diversity. This is particularly evident in bacterial biofilms and can intuitively be attributed to the various gradients of environmental conditions within the different compartments of the community (Stewart and Franklin 2008). However, also under uniform conditions, a bacterial population can develop pronounced heterogeneity (Spudich and Koshland 1976). In general, phenotypic heterogeneity provides a benefit to the population in that it establishes subpopulations that are better prepared for unforeseen changes in conditions (a bet-hedging strategy) and by enabling a division of labor by cooperating cells. This topic has been covered by a number of excellent reviews (Veening et al. 2008; Ackermann 2015; Grimbergen et al. 2015; West and Cooper 2016; Schröter and Dersch 2019).

Phenotypic heterogeneity similarly occurs in c-di-GMP signaling and benefits the whole population, as has been shown, e.g. during biofilm formation of *Escherichia coli* (Serra et al. 2015; Klauck et al. 2018; Serra and Hengge 2019), survival of *Salmonella enterica* serovar Typhimurium in macrophages (Petersen et al. 2019) or, under more uniform conditions, during surface sensing and spread of infection caused by *Pseudomonas aeruginosa* (Armbruster et al. 2019; Laventie et al. 2019). Generally, phenotypic heterogeneity may arise from stochasticity (Elowitz et al. 2002), bistability (Dubnau and Losick 2006), and/or phase variation [e.g. initiated by epigenetic modifications (Casadesús and Low 2006)]. In addition, heterogeneity can be generated upon unequal distribution of proteins with low abundance or inherent polarity during asymmetric cell division (Elowitz et al. 2002; Dworkin 2009).

In several bacterial species, it has been demonstrated that enzymes with DGC or phosphodiesterase (PDE) activity are localized to the cell poles. Generally, polar asymmetric distribution occurs for numerous bacterial proteins and protein complexes, which are involved in many important or even critical cell processes such as DNA segregation, cell division, motility, and others (Laloux and Jacobs-Wagner 2014; Treuner-Lange and Søgaard-Andersen 2014). In the following, we will review what is known about the enzymes and function of polarly localized c-di-GMP production or degradation in the three model species *Caulobacter crescentus, P. aeruginosa*, and *Shewanella putrefaciens*.

### Caulobacter crescentus

Polar organization of c-di-GMP signaling has been best studied in the context of the cell cycle in the alphaproteobacterium *C. crescentus* (Jenal et al. 2017). Cells of this species exist in two distinct morphological states. One is the motile swarmer cell that is monopolarly flagellated and piliated. The swarmer cell remains replicationally quiescent and non-dividing until differentiating into the second state, the stalked cell, by shedding the flagellum and developing a cell extension, the stalk, at the same pole. An adhesion complex located at the tip of the stalk, the so-called holdfast, mediates tight interaction with a substratum. In contrast to the motile swarmer cell, this sessile stalked cell is competent for replication. Cell division occurs in a highly asymmetric fashion, giving rise to a new flagellated swarmer cell, while the stalked daughter cell will remain attached and is able to immediately start a new replication cycle (Kirkpatrick and Viollier 2012; Tsokos and Laub 2012).

c-di-GMP has been shown to affect *C. crescentus* pole morphogenesis and cell cycle control (Jenal et al. 2017). This occurs by fluctuating levels of c-di-GMP during the different stages of the cell cycle (Abel et al. 2013; Lori et al. 2015), which could be impressively demonstrated by a FRET-based approach that allowed visualization of the levels of c-di-GMP within a single cell (Christen et al. 2010). Estimations of the cellular c-di-GMP levels indicate a transient low concentration (<100 nM) in cells arrested in the G1 phase, e.g. the swarmer cells, which increases at least about 3-fold when the cell enters the S-phase (Christen et al. 2010; Abel et al. 2013). The cellular response to the fluctuating levels of c-di-GMP is mediated through intricate mechanisms involving different effectors (reviewed in Hallez et al. 2017; Jenal et al. 2017). Prominent examples are the c-di-GMP adaptor proteins PopA and TipF. PopA, localized to the swarmer pole by the polar hub protein PodJ and to the stalked cell pole by the hub protein PopZ (Wang et al. 2021), acts as a ClpXP protease adaptor. Upon c-di-GMP binding, PopA stimulates the degradation of, e.g. CtrA, a key cell cycle regulator that silences DNA replication initiation in the swarmer cell at the swarmer-to-stalked-cell transition. Concomitantly, c-di-GMP also directly binds to the cognate kinase of CtrA, CckA, and stimulates its phosphatase activity, which rapidly dephosphorylates and inactivates CtrA (Duerig et al. 2009; Ozaki et al. 2014; Smith et al. 2014). In contrast, TipF is involved in initiating flagella formation. c-di-GMP binding stabilizes TipF, which is then localized to the non-stalked cell pole by the landmark protein TipN to where it recruits the first flagellar building blocks (Davis et al. 2013). Recent studies showed that c-di-GMP also directly stimulates the kinase SkhA, another major cell cycle protein whose c-di-GMP binding at the onset of cell differentiation is required for the G1-to-S transition (Dubey et al. 2020; Kaczmarczyk et al. 2020).

*Caulobacter crescentus* possesses 14 GGDEF/EAL domain proteins. Among those, the DGCs DgcB, PleD, and the PDE PdeA have been shown to be active c-di-GMP-synthesizing and -degrading proteins, which play major roles in c-di-GMP-dependent regulation of the cell cycle. Within the scope of this review, we will primarily consider the latter two proteins, the DGC PleD and the PDE PdeA, as they are subject to highly original spatiotemporal regulation at the cell poles.

**Figure 1.**
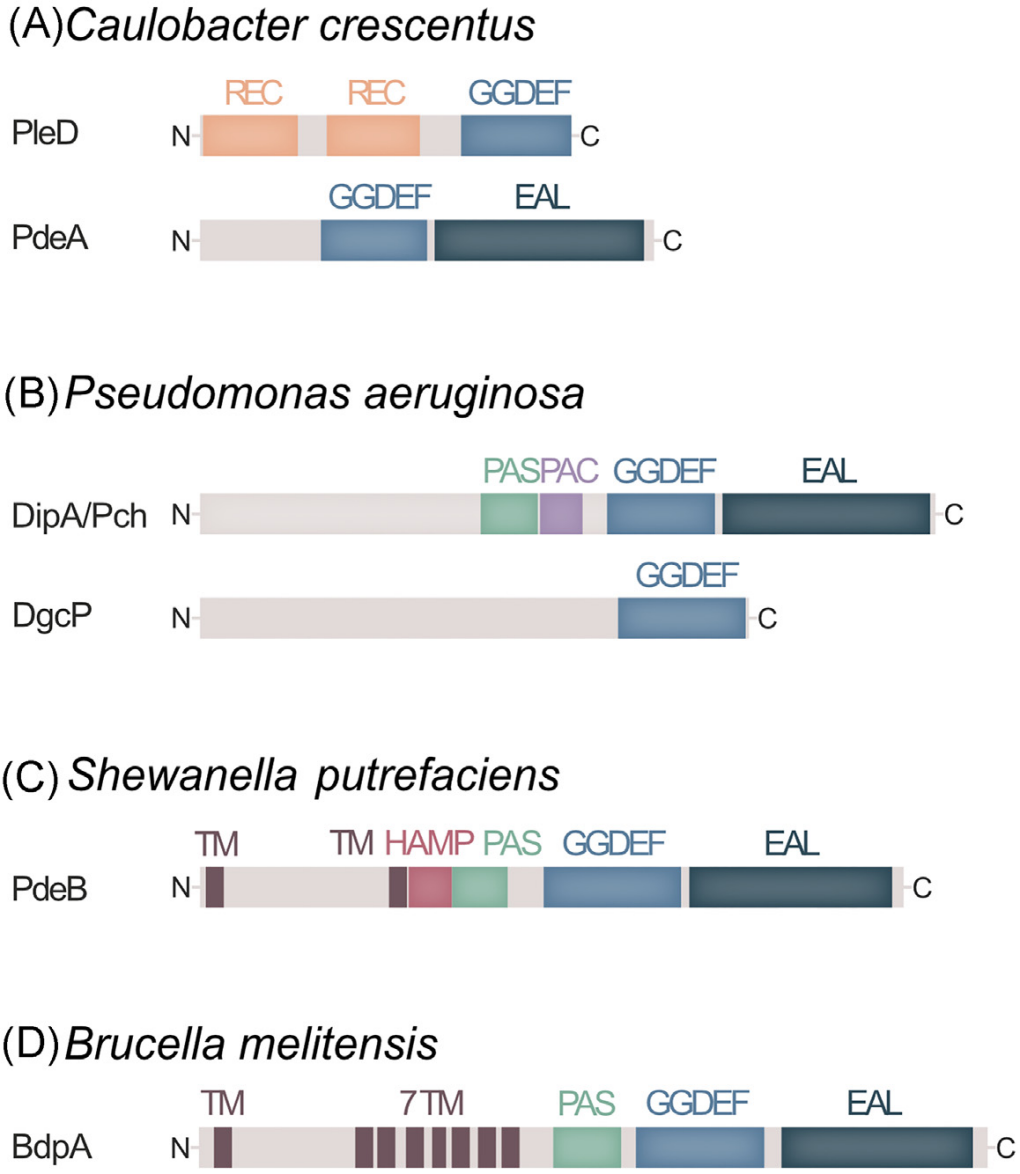
Domain organization of polarly located DGC and phosphodiesterases. **(A)** In *C. crescentus*, the DGC PleD and the PDE PdeA are polarly localized to the stalked and flagellated cell poles, respectively. PleD activity and localization are mediated by phosphorylation of the N-terminal REC domain, PdeA can be actively degraded. **(B)** In *P. aeruginosa*, the PDE Pch is directly or indirectly recruited to the flagellated pole by the chemotaxis protein CheA. The further domains may have a role in signal perception or interaction with other, as yet unknow, client proteins. The DGC DgcP may directly interact with the polar landmark protein FimV via its GGDEF domain, which is thought to activate the DGC activity. (C) *Shewanella* PDE PdeB is recruited by the landmark protein HubP and gets activated through direct interaction. The periplasmic region and the PAS domain are likely involved in signal perception. (D) In *Brucella melitensis*, the PDE BdpA is localized to the cell pole; a homologous PDE, RgsP, is present in *Sinorhizobium meliloti* and other Rhizobiales. BdpA and RgsP are localized within the cytoplasmic membrane by a 7 transmembrane domain (7TM). Potential signals activating the polar PDE activity as well as a phenotypic output are so far unknown.

**Figure 2.**
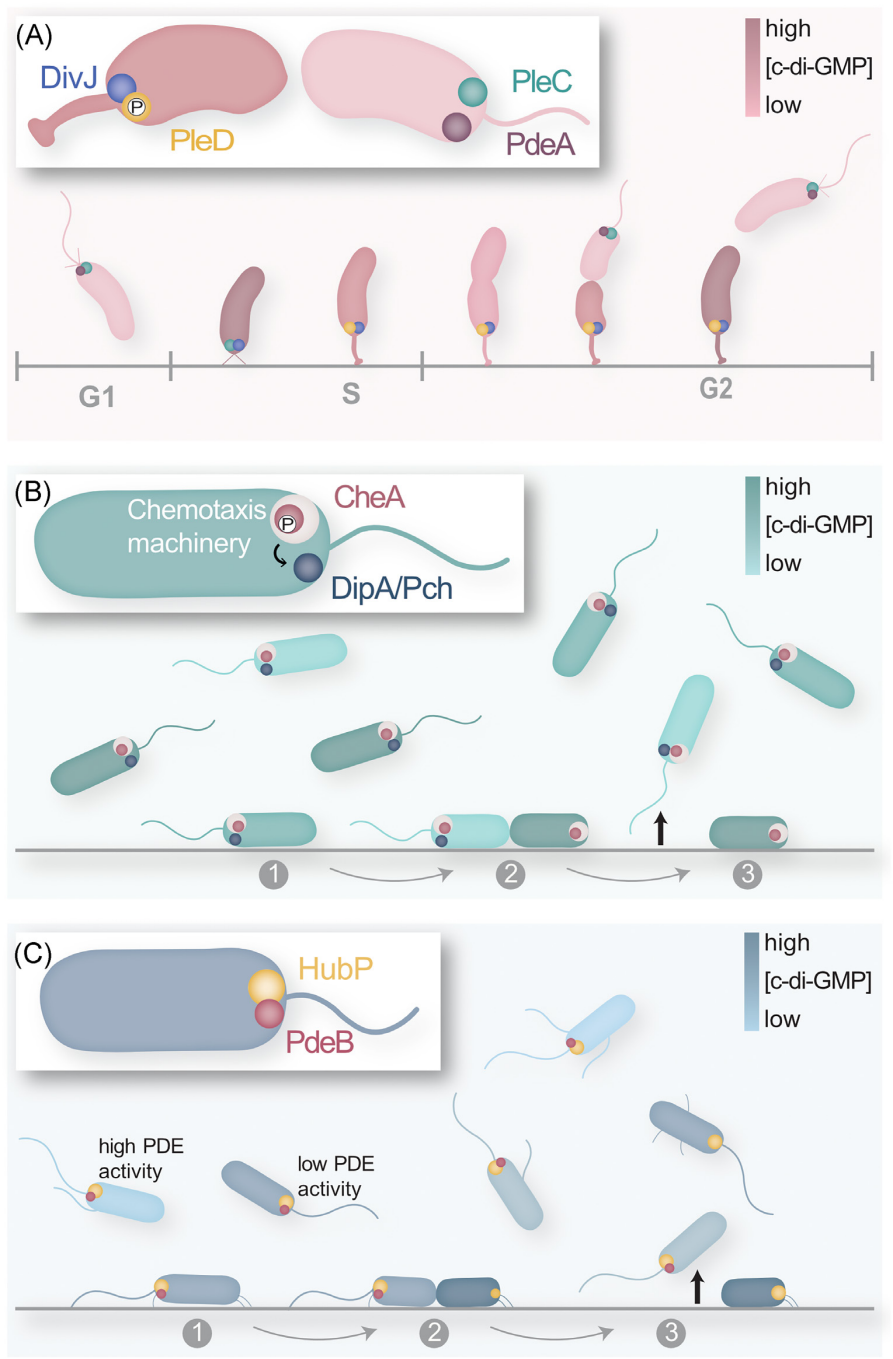
Localization pattern of PDEs and DGCs. **(A)** During the *C. crescentus* cell cycle, DGC PleD gets phosphorylated and thereby polarized by DivJ at the stalked cell pole. PleC at the opposite flagellated cell pole dephosphorylates and inactivates PleD. The antagonistic PDE PdeA is also located at the flagellated cell pole to keep c-di-GMP levels low. PdeA gets degraded once the flagellated cell pole becomes the stalked cell pole. The activity of the proteins results in the observed oscillations in c-di-GMP levels during the cell cycle. The cell-cycle phases are indicated below. **(B)** The PDE Pch of *P. aeruginosa* directly or indirectly interacts with the chemotaxis histidine kinase CheA at the flagellated cell pole. Activity of Pch depends on the phospohorylation state and hence on the chemotactic signals the cell perceives. The asymmetry and heterogeneity in c-di-GMP degradation results in a highly heterogeneous population where the individual cells are differently primed for surface association. The asymmetric cell division with PdeB at the flagellated pole in surface-associated cells (1) results in two progeny cells with low and high c-di-GMP levels, respectively (2). The flagellated and highly motile mother cell frequently detaches again (3). This behavior mediates efficient spreading in the environment. **(C)** The *Shewanella* PDE PdeB is recruited through direct interaction with the landmark protein Hub; this interaction is required for the general activity of PdeB. The signals perceived are as yet unknown. Pch leads to a pronounced heterogeneity in c-di-GMP within the global population (see also Fig. 3), which also due to the differences in the number of PdeB copies per cell. The PdeB-mediated asymmetry during cell division of surface-associated cells similarly enables the ‘Touch-Seed-and-Go’ behavior as shown for *P. aeruginosa*. The levels of c-di-GMP are illustrated by color intensities as indicated in the respective panels.

PleD is a response regulator that harbors two consecutive receiver (REC) domains in its N-terminal region and a GGDEF output domain at its C-terminus (Hecht and Newton 1995) (Fig. 1A). Loss of PleD results in cells that fail to efficiently produce a stalk, they do not eject their flagellum and remain motile during the cell cycle (Sommer and Newton 1989; Aldridge and Jenal 1999). The DGC activity was found to be essential for the overall function of the protein, and a constitutively active PleD variant substantially decreased flagella-mediated motility (Aldridge et al. 2003). How is spatial activity of PleD achieved in cells of *C. crescentus*? Phosphorylation at an aspartate residue within the upstream region of the two PleD N-terminal REC domains leads to PleD dimerization and activation (Paul et al. 2007). Two cognate kinases, DivJ and PleC, directly interact with PleD and affect its phosphorylation state (Aldridge et al. 2003; Paul et al. 2004). Notably, both kinases are located at opposite cell poles: DivJ clusters at the stalked cell pole, while PleC localizes to the flagellated and piliated cell pole of swarmer cells (Wheeler and Shapiro 1999) (Fig. 2A). Of the two, DivJ functions as a kinase for PleD and keeps the phosphorylation and thus the DGC activity level high. In addition, phosphorylated PleD remains localized with DivJ at the stalked cell pole. In contrast, PleC acts as a phosphatase on PleD and shuts down its DGC activity (Paul et al. 2004). Thus, by specific localization of the two antagonizing kinases, PleD DGC activity is restricted to the stalked cell pole. Notably, it is not yet clear how phosphorylated dimeric PleD is recruited to the designated pole. When co-expressed in *E. coli*, PleD exhibited partial co-localization with PopZ, a polarity factor serving in *C. crescentus* as a hub to localize many proteins at the pole (Holmes et al. 2016). However, PleD localization occurred normally in *C. crescentus* mutants lacking PopZ or any other of the known polarity proteins TipN, TipF, SpmX, or PodJ (Ozaki et al. 2014). Thus, active PleD may be recruited by more than one of these polar markers or additionally or separately by a yet unknown polarity factor.

In addition to the DGC activity of PleD, also PDE activity is localized accordingly: The phosphodiesterase PdeA (Fig. 1A) is recruited to the flagellated (PleD-free) cell pole, where it assists in keeping the c-di-GMP concentration low (Fig. 2A). At the stalked cell pole, PdeA is actively degraded by the ClpXP protease (Abel et al. 2011), which in turn is activated by high c-di-GMP levels via the ClpXP-adaptor protein PopA (see above). Thus, PopA links cell cycle progression and morphological differentiation to c-di-GMP levels through the proteolysis of key effectors, e.g. CtrA and PdeA. The effective spatiotemporal localization of the antagonizing DGC and PDE activities to the opposite cell pole results in the observed oscillation of c-di-GMP levels (Christen et al. 2010; Abel et al. 2013; Lori et al. 2015), which ultimately gives rise to the two morphologically distinct offspring.

### Pseudomonas aeruginosa

The gammaproteobacterium *P. aeruginosa* has emerged as one of the model species to study c-di-GMP in bacterial signaling. Compared to *C. crescentus*, there is no obvious effect on the cell cycle by c-di-GMP in this species. Accordingly, studies on this second messenger in *P. aeruginosa* have predominantly addressed its role in biofilm formation and pathogenicity. As in most other bacteria, a high level of c-di-GMP favors a sessile rather than a planktonic lifestyle. *Pseudomonas aeruginosa* encodes a plethora of proteins with potential DGC/PDE activities, and a number of c-di-GMP effector proteins have been identified that affect different aspects of cell physiology (Valentini and Filloux 2019; Lichtenberg et al. 2022; Park and Sauer 2022).

The aforementioned study by Christen et al. (2010), which applied a FRET-based reporter system to visualize c-di-GMP levels, showed that also *P. aeruginosa* cell division gives rise to daughter cells with a bimodal distribution of c-di-GMP levels during exponential growth. That was a surprising find as *P. aeruginosa* produces offspring cells that are not as obviously different as the stalked and swarmer cells of *C. crescentus*. Among the 38 proteins with potential DGC/PDE activity in *P. aeruginosa* PAO1, mutants lacking just a single protein, named Pch, lost the bimodal c-di-GMP distribution. Also, the rather broad range of c-di-GMP levels observed for single cells within the population (Fig. 2B) was drastically shifted toward a narrow range with high concentrations (>600 nM as determined by the FRET reporter) (Kulasekara et al. 2013). Pch (formerly DipA) is a cytoplasmic GGDEF/EAL hybrid protein with a GAF and a PAS domain at its N-terminus (Fig. 1B), which acts as a PDE (Roy et al. 2012). *Pseudomonas aeruginosa* mutants lacking Pch exhibit altered chemotactic behavior and lowered cell velocity, reduced spreading ability through soft agar, impaired swarming ability, but enhanced initial attachment and increased biofilm formation (Li et al. 2007; Roy et al. 2012; Kulasekara et al. 2013).

Pch localizes to the flagellated cell pole and is directly or indirectly recruited by CheA of the flagellum-associated polar chemotaxis system in *P. aeruginosa* (Kulasekara et al. 2013). CheA is the central histidine kinase in the signaling cascade within the large chemotaxis machinery, which is also located at the flagellated pole (see Muok et al. 2020; Colin et al. 2021). While it is still unclear how Pch and CheA interact, they form a complex, and, notably, the PDE activity of Pch depends largely on the phosphorylation level of CheA (Kulasekara et al. 2013). *Pseudomonas aeruginosa* possesses a large array of 26 MCPs, indicating that a wide range of external signals can be perceived, which is thus elegantly linked via CheA to the activity of Pch. Intuitively, this activity will be highest (and hence the c-di-GMP level lowest) under conditions that also trigger frequent switches of flagellar rotation, e.g. when cells are swimming down a gradient of attractants. It will be interesting to further explore which chemotactic signals (or which signal combination) effectively mediate Pch activity, if the observed pronounced heterogeneity of c-di-GMP levels is mediated by the chemotaxis input, and how the c-di-GMP signaling with its multiple effects on chemotaxis impacts the bacterial lifestyle (Colin et al. 2021; Keegstra et al. 2022).

By restricting Pch to the flagellated cell pole, one daughter cell inherits a fully functional flagellum along with the chemotaxis system associated with Pch upon cell division, which keeps the cellular c-di-GMP level generally low. In contrast, the second daughter cell does not yet possess a functional flagellum and lacks Pch. In this way, a planktonic population of *P. aeruginosa* exhibits a broad distribution of cellular behaviors even under uniform conditions, ranging from fast swimming planktonic cells to cells primed for attachment to surfaces. A more recent study nicely demonstrated that the asymmetric cell division affects the surface colonization of *P. aeruginosa* (Laventie et al. 2019). Surface attachment of a cell is followed by instant increase of c-di-GMP levels, which leads to dimerization of the c-di-GMP-binding effector protein FimW. FimW is recruited to the cell poles where it stimulates the assembly of type IV pili. Asymmetric cell division at the surface causes delocalization of FimW after cell fission from the cell pole that also harbors Pch due to the lower c-di-GMP levels. This ‘mother’ cell, equipped with a functional flagellum and chemotaxis system but less active pili, would then frequently detach from the surface, leaving behind the still surface-associated ‘daughter’ cell (Laventie et al. 2019) (Fig. 2B). Remarkably, spreading assays on epithelial lung cell layers demonstrated that the asymmetric cell division followed by detachment of the ‘swarmer’ cell benefits tissue colonization and host cell damage (Laventie et al. 2019). It needs to be mentioned that, in addition to CheA/Pch, also the c-di-GMP-synthesizing Wsp system contributes to the heterogeneity of c-di-GMP levels in surface-associated cells (Armbruster et al. 2019). Notably, when activated, the components of this system also form distinct clusters at the cell envelope (Güvener and Harwood 2007), which demonstrates that Wsp DGC activity is spatially regulated to some degree, albeit not at the cell poles.

Another protein involved in c-di-GMP production, the DGC DgcP, has recently been demonstrated to be polarly localized in *P. aeruginosa* (Nicastro et al. 2020). DgcP is a cytoplasmic protein with a C-terminally located GGDEF domain but no additional domains with predic functions (Fig. 1B). Loss or overproduction of the protein affects pathogenicity, cell fitness upon exposure to imipenem, twitching motility, and biofilm formation (Nicastro et al. 2014, 2020; Aragon et al. 2015). In contrast to Pch, DgcP is recruited to the cell poles by the polar landmark protein FimV through direct contact with the GGDEF domain of DgcP. Notably, this interaction with FimV appears to stimulate the DGC activity of DgcP (Nicastro et al. 2020). The mechanistic role of DgcP, e.g. in type IV pili synthesis, is not yet clear. The localization studies (based on overproduction of the fluorescently labeled protein) suggest a bipolar localization pattern, which may hint at a role for DgcP as an antagonistic DGC for Pch at the flagellated and non-flagellated cell poles; however, this remains to be shown in future experiments.

### *Shewanella* species

Polar localization of c-di-GMP regulation has been studied in more detail also in the gammaproteobacteria *Shewanella* spp. While there are as yet no indications that c-di-GMP is involved in cell cycle regulation in members of this genus, c-di-GMP heavily affects the switch between planktonic and sessile life styles (Thormann et al. 2006). *Shewanella oneidensis* and *S. putrefaciens* bear a high number of proteins with potential DGC and/or PDE activity (51 in *S. putrefaciens*). However, only the role of few of them has been characterized yet, of which one is PdeB. PdeB is a transmembrane hybrid GGDEF/EAL domain protein that, in addition, harbors a putative periplasmic sensor domain and cytoplasmic HAMP and PAS domains (Fig. 1C). PdeB exclusively functions as a PDE in response to environmentally complex nutrients, as it is present but inactive in defined mineral media (Chao et al. 2013; Rossmann et al. 2019). Loss of the protein results in a two-fold increase in c-di-GMP levels within a bulk planktonic population, a decrease in flagella-mediated motility, and in increased abundance and activity of surface adhesion proteins and the MSHA type IV pili (Chao et al. 2013; Rossmann et al. 2019; Rick et al. 2022). PdeB is recruited to the flagellated cell pole by the polar landmark protein HubP, a homolog of *Pseudomonas* FimV. The tight interaction between both proteins is mediated by the inactive GGDEF domain of PdeB. Notably, PdeB activity strictly requires interaction of its GGDEF domain with its landmark HubP, which therefore acts as a spatial on-switch within the cell. To enable recruitment and activation, the structure of the GGDEF domain had evolved accordingly and other, canonical GGDEF domains cannot be recruited (Rick et al. 2022). Of note, the GGDEF domain of *P. aeruginosa* DgcP, which mediates recruitment of DgcP by the landmark protein FimV through direct interaction (Nicastro et al. 2020; see above), exhibits a structure quite distinct from that of GGDEF_PdeB_, thus implicating a different mode of interaction and activation (Rick et al. 2022).

**Figure 3.**
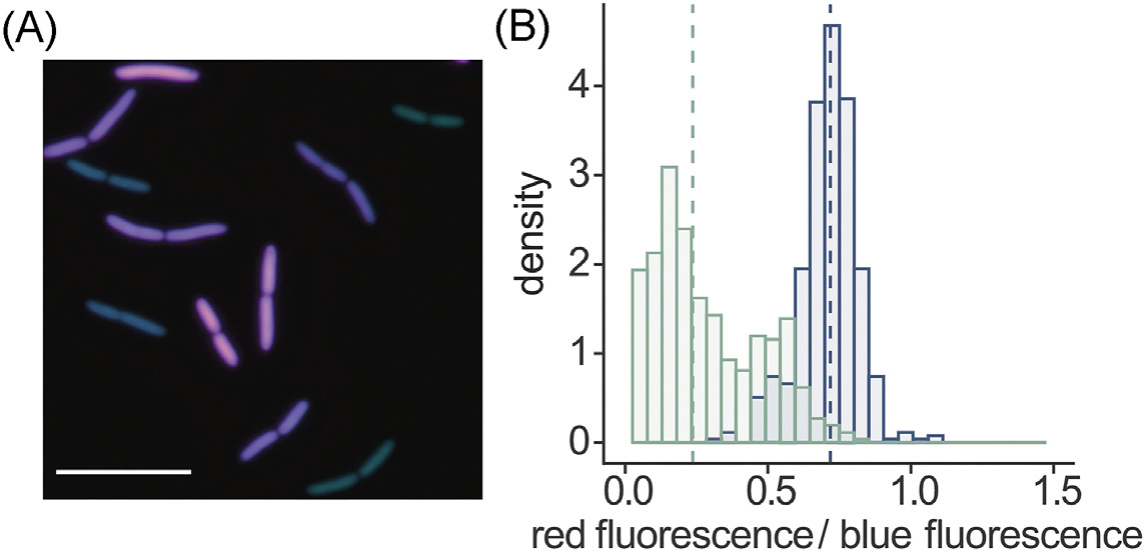
PdeB-based heterogeneity in c-di-GMP levels in *S. oneidensis*. Asymmetric distribution in concert with varying timing and amount of PdeB production results in a pronounced heterogeneity of c-di-GMP levels in a planktonic *S. oneidensis* population. **(A)** Micrograph of *S. oneidensis* cells bearing a fluorescence reporter based on a c-di-GMP-dependent riboswitch. The scale bar equals 10 µm. **(B)** The diagram shows the distribution of c-di-GMP-dependent fluorescence normalized to the plasmid copy number over a planktonic population of *S. oneidensis*. The wild type is shown in green, a *ΔpdeB* mutant in dark blue.

The presence and activity of PdeB results in a broad distribution of c-di-GMP levels of the individual cells in planktonic cultures under uniform conditions (Fig. 3). This heterogeneity in c-di-GMP content likely emerges from the non-uniform timing of PdeB production, a strong variation in the predominantly low number of individual PdeB molecules (0–16 molecules per cell), and the bimodal cell division due to the monopolar positioning of PdeB. Thus, even under uniform conditions, PdeB induces heterogeneity in cellular behavior and differentially primes the cells for surface attachment. Once surface-associated, the polar PDE activity also enables *S. putrefaciens* cells to split the daughter cells into a swarmer and a surface-associated cell as described for *P. aeruginosa* (Laventie et al. 2019; see above) (Fig. 2C), which benefits the expansion and the proliferation of the population within the environment (Rick et al. 2022). Open questions regarding the *Shewanella* HubP/PdeB system remain, particularly concerning the identity of the external signal(s) to which PdeB responds and the regulation of the amount and timing of PdeB production, which, potentially due to stochastic effects arising from its low copy number, has a high impact on the phenotypic heterogeneity of c-di-GMP signaling.

## Outlook and conclusions: lots of unknowns

### How common is polar localization of c-di-GMP synthesis/degradation?

The given examples of *C. crescentus, P. aeruginosa*, and *Shewanella* ssp. illustrate the different mechanisms by which bacteria realize effective polarity with respect to c-di-GMP signaling. But how widespread is this phenomenon? There are numerous indications and some direct experimental evidence that polar localization of DGCs or PDEs is not restricted to a few bacterial species. The most important lead is that all systems described here have clear homologs in other bacteria. Parts of the intricate cell cycle regulation machinery that are active in *C. crescentus* also appear in other alphaproteobacteria (see Hallez et al. 2017). Whether this also involves active DGCs/PDEs and oscillations of c-di-GMP levels over the cell cycle remains to be shown.

Studies on c-di-GMP regulation in *S. meliloti* identified a homolog to PleD that exhibits polar localization, albeit in a temporal pattern different from that of *C. crescentus*, suggesting that polarity of c-di-GMP regulation may also play a role in *S. meliloti* (Schäper et al. 2016). In the same species, the PDE RgsP (Fig. 1D) was found to localize to the flagellated pole. However, the activity of RgsP as a PDE does not appear to be required for the function of the protein in unipolar growth and thus remains unknown as for the time being. Homologs to RgsP are widespread among the Rhizobiales (Schäper et al. 2018). In addition, a homolog to RgsP was identified and characterized in *B. melitensis*, here named BdpA (Petersen et al. 2011). As in *S. meliloti*, BdpA localizes to the cell pole, but the PDE activity seems to be dispensible for BdpA function in mediating growth and cell morphology, at least not under the conditions tested (Reboul et al. 2021). Further studies are required to elucidate the role of the polar PDE activity in these species.

Also, searches for potential orthologs of *P. aeruginosa* Pch or DgcP readily identify numerous potential functional orthologues, at least among *Pseudomonas* ssp. A function for DgcP has already been demonstrated for another species of *Pseudomonas, P. savastanoi*; however, there is no experimental evidence for polar localization of the protein in this species yet (Aragon et al. 2015). The corresponding recruiting landmark proteins, CheA for Pch and FimV for DgcP, are also widely conserved within the pseudomonads.

In *Shewanella* ssp., the GGDEF domain of PdeB has evolved toward an interaction partner for the C-terminal FimV domain of the polar landmark protein HubP/FimV (Rick et al. 2022). Proteins harboring PdeB-like GGDEF domains regularly include residue variants that may mediate interaction with the highly conserved FimV domain of polar landmark HubP proteins are present in numerous different bacteria, e.g. in diverse *Vibrio* species, which also harbor a clear HubP ortholog (Yamaichi et al. 2012; Altinoglu et al. 2022). These proteins are good candidates to examine the polar localization and asymmetric distribution of proteins and c-di-GMP levels upon cell division. Finally, the gammaproteobacteria *S. enterica* serovar Typhimurium and *Klebsiella pneumonia*, which exhibit peritrichous flagellation patterns, lack polar landmark proteins like HubP/FimV. Nonetheless, these displayed asymmetric distribution of c-di-GMP (Christen et al. 2010). How this occurs and whether this is due to the polar localization of the corresponding DGCs or PDEs is not clear.

### Is there local polar signal perception?

Numerous studies showed that c-di-GMP (and hence enzymes with DGC and PDE activity) affect flagella assembly and function, chemotaxis, and pilus activity (Wolfe and Visick 2008; Jenal et al. 2017; Laventie and Jenal 2020). On the other hand, flagella and pili have been implicated as environmental sensors, which are involved, e.g. in surface sensing as shown for *C. crescentus, Vibrio* ssp., *P. aeruginosa*, and others (McCarter et al. 1988; Ellison et al. 2017; Hug et al. 2017; Laventie et al. 2019; Schniederberend et al. 2019; Floyd et al. 2020; Laventie and Jenal 2020; Hershey et al. 2021; Koch et al. 2022; Matilla et al. 2022). While flagellar motor-stator remodeling has been shown to be contribute to c-di-GMP production in *P. aeruginosa* (Kuchma et al. 2015; Baker et al. 2016, 2019), it is still mostly elusive how bacterial cells translate disturbances such as complete stops or forced slowing of flagellar rotation or increase in pilus tension into an increase in c-di-GMP levels. While not essential, it may be advantageous in polarly flagellated (or piliated) species to place the corresponding DGCs or PDEs close to the sensor complexes. Whether Pch, PleD, or PdeB are directly involved in rapid surface sensing is not yet clear and a question that is not easy to tackle as these proteins require their polar localization for normal function. It is also conceivable that other, yet unidentified, polar PDE/DGC proteins or their effectors are involved.

## Conclusions

Taken together, these examples imply that the polarity of c-di-GMP synthesis and degradation is widespread among bacteria, and that many systems remain to be explored. The development of further fluorescence-based reporters sui for single cell analysis of c-di-GMP levels in Gram-negative as well as Gram-positive cells (Rybtke et al. 2012; Borlee et al. 2017; Petersen et al. 2019; Weiss et al. 2019; Zamorano-Sánchez et al. 2019) will help to characterize these systems, the underlying regulatory and recruitment mechanisms, and their role in bacterial proliferation, surface association, spread, and pathogenicity.
